# Cryptococcus meningitis mimicking cerebral septic emboli, a case report series demonstrating injection drug use as a risk factor for development of disseminated disease

**DOI:** 10.1186/s12879-020-05108-1

**Published:** 2020-05-27

**Authors:** Christopher Polk, Jacqueline Meredith, Alyssa Kuprenas, Michael Leonard

**Affiliations:** 1 Atrium Health Department of Medicine, Charlotte, NC USA; 2 Atrium Health, Department of Pharmacy, Antimicrobial Support Network, Charlotte, NC USA

**Keywords:** Cryptococcus meningitis case report, *Cryptococcus neoformans*, Intravenous drug use, Central nervous system vasculitis

## Abstract

**Background:**

Clinicians may be less inclined to consider a diagnosis of cryptococcal meningitis in people without HIV infection or transplant-related immunosuppression. This may lead to a delay in diagnosis particularly if disseminated cryptococcal disease mimics cerebral septic emboli in injection drug use (IDU) leading to a search for endocarditis or other infectious sources. Though, IDU has been described as a potential risk for disseminated cryptococcal disease.

**Case presentations:**

We present two cases of cryptococcal meningitis in IDU without HIV or other obvious immune deficits. Both patients presented with at least 2 weeks of headache and blurred vision. They developed central nervous system (CNS) vasculitis, one of which mimicked septic cerebral emboli, but both resulted with poor neurologic outcomes.

**Conclusions:**

IDU likely induces an underappreciated immune deficit and is a risk factor for developing cryptococcal meningitis. This diagnosis, which can mimic cerebral septic emboli through involvement of a CNS vasculitis, should be considered in the setting of IDU.

## Background

While physicians may be familiar with the presentation of cryptococcal meningitis in people living with HIV, clinicians may be less inclined to consider the diagnosis in other patient populations leading to a delay in diagnosis. Appreciation of non-HIV risk factors for cryptococcal disease may be helpful for physicians, with risk for cryptococcal disease described in organ transplant recipients, patients with hematologic malignancies, sarcoid, liver disease, or receiving immunosuppressive agents [1, 2]. A few prior case reports have also suggested that injection drug use (IDU) even without concomitant HIV disease may be a risk factor for cryptococcal meningitis [2, 3]. We describe two cases of cryptococcal meningitis in the setting of IDU without HIV to corroborate previous reports of increased risk of cryptococcal disease in this patient cohort. Particularly, we note that cryptococcal meningitis may lead to a vasculitis and mimic septic cerebral emboli that is more commonly found in IDU-associated endocarditis, which may subsequently confound clinicians in considering cryptococcosis as a diagnosis.

## Case presentations

### Case 1

A 26-year-old male with reported IDU of heroin presented with 2 weeks of headache, blurred vision, and confusion with nonsensical speech without fevers. Lumbar puncture revealed a CSF WBC count of 423 cells/μL, 64% lymphocytes, with RBC 6 cells/μL and a CSF glucose of 26 mg/dL (serum glucose of 139 mg/dL). HIV, hepatitis testing and serum cryptococcal Ag titer returned negative at that time (see Table 1). The patient continued to decline with confusion and garbled speech despite empiric antibiotics and antiviral therapy. On hospital day 10, a repeat LP was obtained (CSF WBC 204 cells/μL; 66% lymphocytes; RBC 4 cells/μL; glucose < 10 mg/dL) and CSF cryptococcal Ag was positive at 1:2560 and cultures grew *Cryptococcus neoformans*. Interestingly, initial CSF cultures from the prior LP remained without growth. He was treated with liposomal amphotericin and flucytosine and required serial lumber punctures with subsequent placement of a lumbar drain to manage his CSF pressures. CSF cultures cleared within 4 days of therapy, but due to lack of clinical improvement steroids were added after 10 days. He improved, completed 6 weeks of induction therapy with liposomal amphotericin and flucytosine, steroids were tapered and a VP shunt placed. He was discharged on fluconazole 400 mg daily for consolidation therapy. Outpatient**,** fluconazole was reduced to 200 mg daily for maintenance therapy 3 weeks later.

**Table 1:** Summary of Patient Cases

	CASE 1	CASE 2
Age	26	30
Gender	Male	Male
Significant comorbidities	–	HCV
HIV status	Negative (HIV RNA not detected)	Negative
CD4, cells (%)	474 (40%)	754 (39%)
Duration of symptoms at hospital presentation	2–3 weeks	1 month
Symptoms	Altered mental status, severe headache, seizures	Altered mental status, headaches, dizziness, blurred vision, blurry/double vision, loss of spatial judgement
Reported illicit drug use	Heroin & cocaine	Heroin
Imaging (MRI/CT)	MRI brain: worsening leptomeningeal disease with increased areas of T2 FLAIR hyperintensity and contrast enhancement involving the surfaces of the brain; large bilateral subacute anterior cerebral artery territory infarcts and an infarct in the left middle cerebral artery territory	MRI brain: multiple acute infarctions of the cerebrum, brainstem, and cerebellum, with associated pathologic enhancement, likely secondary to septic emboli from a central source; evidence of basilar predominant leptomeningitis. CTA brain: Irregular narrowing of the M1 ACA and A2 ACA suggestive of vasculitis
Initial lumbar puncture results	Opening pressure 34 mm H_2_O Glucose 26 mg/dL Protein 101 mg/dL RBC 6 cells/μL WBC 423 cells/μL 17% segs, 11% monocytes, 64% lymphocytes	Opening pressure not reported, EVD already in place *Results reported as LP (EVD):* Glucose 12 mg/dL (52 mg/dL) Protein 200 mg/dL (47 mg/dL) RBC 1 cells/μL (195 cells/μL) WBC 34 cells/μL (18 cells/μ) 63% segs, 36% lymphocytes
CSF CrAg	1:2056	> 1:2560 (LP); 1:320 (EVD)
Initial Serum CrAg	Negative	> 1:2560
Species	*Cryptococcus neoformans*	*Cryptococcus neoformans* from lumbar CSF

Six weeks following hospital discharge, the patient was readmitted for worsening headache and confusion. Repeat lumbar puncture on readmission returned with a CSF WBC of 427 cells/μL (80% lymphocytes), RBC 126 cells/μL with a CSF glucose of 14 mg/dL. Cryptococcal Ag remained positive at 1:160, but no organisms were isolated on these or multiple subsequent CSF cultures. He had his VP shunt removed and was again treated with 6 weeks of liposomal amphotericin and flucytosine with methylprednisolone added for 7 days followed by a steroid taper due to concern for a Post-Infectious Inflammatory Response Syndrome (PIIRS). CSF pressures were also managed by serial lumbar punctures and a ventricular drain followed by repeat VP shunt placement. Despite these efforts, he clinically deteriorated with worsening neurologic function and evidence of cerebral vasospasm of the right middle cerebral artery (MCA) and anterior cerebral artery (ACA) on transcranial doppler exam. A repeat MRI brain was obtained that revealed large bilateral subacute ACA territory infarcts and an infarct in the left MCA territory. After 6 weeks of treatment he was transitioned to consolidation therapy with fluconazole 800 mg daily but remains with profound neurologic deficits.

### Case 2

**Fig. 1:**
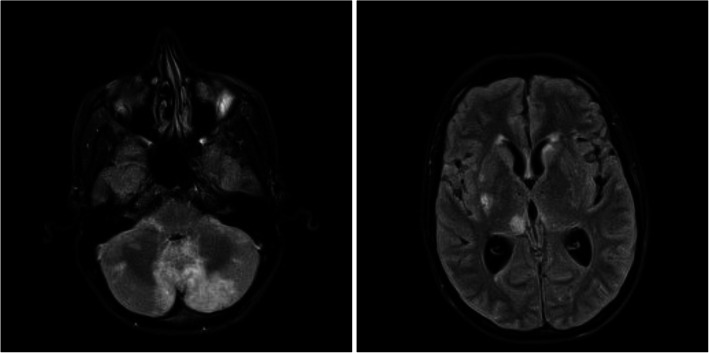
MRI Brain case 2 with multiple acute infarctions in cerebrum, brainstem, and cerebellum read as likely secondary to septic embolic

**Fig. 2:**
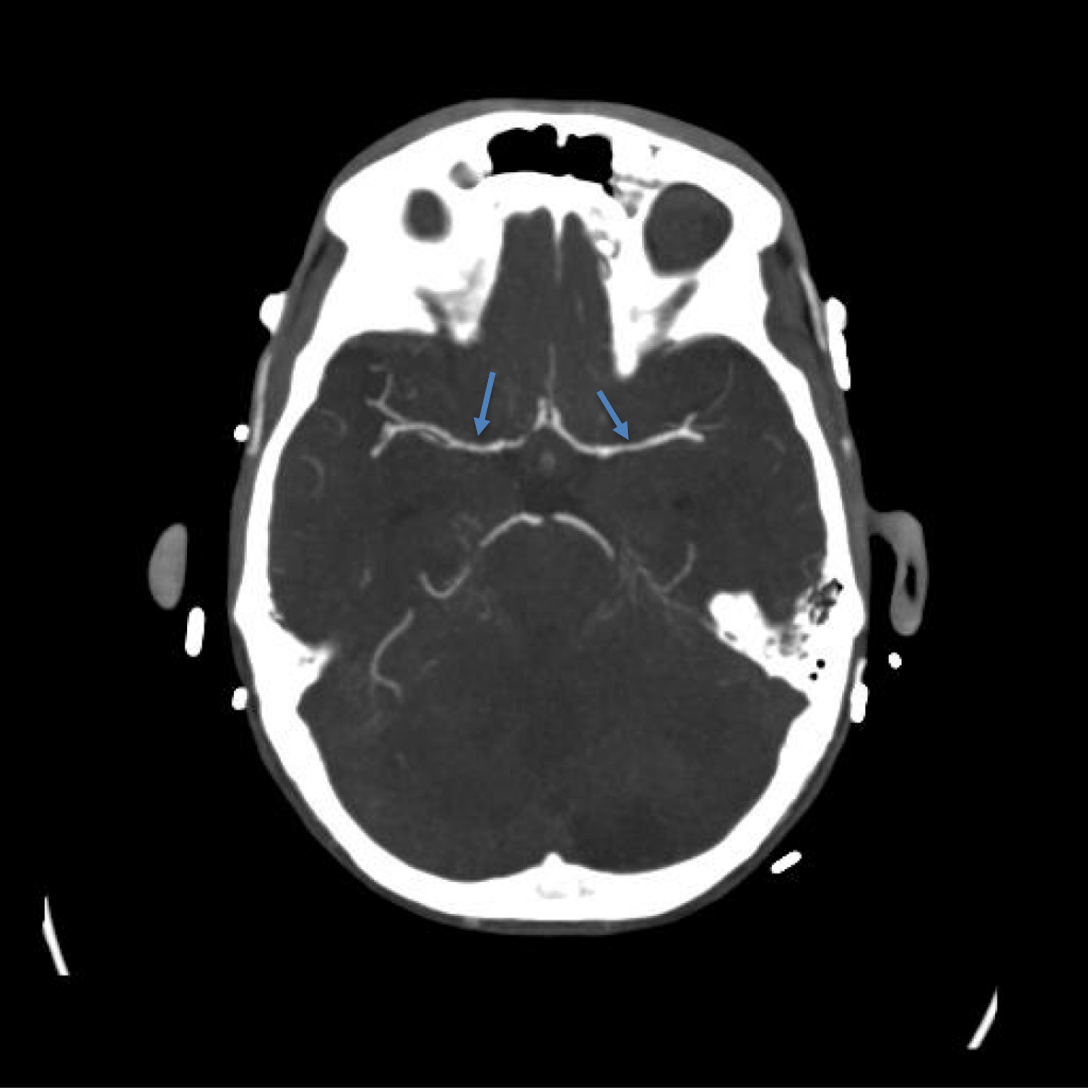
CT Angiogram of brain case 2 revealing irregular narrowing of the M1 MCA consistent with a vasculitis

A 30-year-old male with ongoing IDU and chronic HCV presented with 4 weeks of headache, blurry vision, hearing loss, and gait imbalance. The patient reported a couple of ED visits at an outside hospital the few weeks prior but was sent home each time. CT brain revealed a posterior fossa tumor versus abscess with hydrocephalus. A ventricular drain was placed to manage his hydrocephalus with CSF revealing WBC 18 cells/μL (51% segmented cells), RBC 195 cells/μL and a CSF glucose of 52 mg/dL in context of serum glucose of 119 mg/dL. Cultures were without growth. MRI brain was obtained which revealed multiple acute infarctions in cerebrum, brainstem, and cerebellum likely secondary to septic embolic, but also with basilar leptomeningitis (see Fig. 1). He was initially treated with antibiotics due to concern for endocarditis; however, blood cultures, as well as a TEE were unremarkable. A lumbar puncture was then obtained with CSF WBC 34 cells/μL (63% segmented cells), RBC 1 cells/μL and a CSF glucose of 12 mg/dL. Cryptococcal Ag titers from lumbar CSF obtained returned at 1:2560. CT angiogram was also obtained which revealed irregular narrowing of the M1 MCA and A2 ACA consistent with a vasculitis (see Fig. 2). The patient was treated with liposomal amphotericin and flucytosine, and no steroids were prescribed despite vasculitis findings. Subsequent CSF cultures cleared but clinically the patient did not improve and ultimately expired.

## Discussion and conclusions

Cryptococcal meningitis can be a diagnostic challenge as illustrated by our case series. We note in our cases serum cryptococcal Ag titers may be negative and ventricular CSF may not be diagnostic. The need for lumbar CSF, which may be discordant with ventricular CSF results, to diagnose basilar meningitis such as cryptococcus has been noted in prior case series [4]. In our second case, cryptococcosis presented with an appearance similar to cerebral septic emboli which might be expected with endocarditis, a common diagnosis in the IDU cohort. However, the cerebral septic emboli in this case and the multifocal cerebral infarcts with vasospasm found in our first case were due to CNS vasculitis. CNS vasculitis is described rarely secondary to cryptococcus, but may be associated with as worse prognosis which our cases demonstrated [5, 6]. Cryptococcal neuro-invasion is mediated through direct trapping of organisms and migration through the capillary wall with such involvement of the vessel wall potentially leading to vasculitis [7]. While steroids are indicated for treatment of CNS vasculitis, whether outcomes in vasculitis secondary to cryptococcus are improved by steroids is uncertain [5, 6, 8]. Our first case received steroids as appropriate therapy due to concern for PIIRS but did not improve [9].

Clinicians should consider cryptococcal infection as a diagnostic consideration in IDU with cerebral disease due to the apparent increased risk of disseminated disease in this cohort. While meningitis caused by Candida or Mucor organisms seen in IDU might be due to direct bloodstream inoculation, the mechanism of infection is not entirely clear with cryptococcal meningitis but we are unaware of evidence to suggest a mechanism of infection aside from inhalation [2, 10]. The increased infection risk could be related to increased exposure to infections, depressed immunity, or a combination of both. We postulate that the risk of cryptococcal disease is increased in IDU due to the immune suppressive effects of heroin and methamphetamine.

An increased incidence and severity of all types of infections has long been noted in IDUs, suggesting impaired immunity [11]. Alterations of both the innate and adaptive immunity have been described in both acute- and long-term opioid administration; several mechanisms have been reported, including alterations of the hypothalamic-pituitary-adrenal axis and the autonomic nervous system with prolonged opioid exposure [12]. Particularly, heroin has been reported to suppress T-lymphocyte responses known to be important in cryptococcal disease. These effects have even been identified in periods of heroin withdrawal [13]. It is postulated that this may have to do with the direct effects of heroin on opiate receptors located on immune cells. Animal studies have suggested that this can result in decreased antibody production, phagocytosis, and cytokine production [14]. In addition, methamphetamine use alters antigen processing and facilitates cryptococcal dissemination from the lung to the brain in animal models [15]. These alterations on the immune system with T-cell mediated deficits may predispose individuals to fungal infections such as cryptococcosis which is suggested in epidemiologic studies of fungal disease prevalence in IDU [10]. In fact, we are aware of at least two other recent cases of fungal meningitis in IDU at our institution. Findings from our case series, as well as a case series by Shorman et al., suggest that IDU is indeed a risk factor for disseminated cryptococcosis infection due to IDU -associated immune suppression [2].

More cases of cryptococcosis are expected in the IDU population with the ongoing opioid epidemic. We urge clinicians to consider a diagnosis of cryptococcal disease in IDUs, which may present as similarly to septic cerebral emboli due to CNS vasculitis or with other atypical manifestations. IDU appears to be a significant risk factor for development of disseminated cryptococcal infection.

## Data Availability

All data are contained within the article.

## Abbreviations

ACA: Anterior cerebral artery
CNS: Central nervous system
CSF: Cerebral spinal fluid
EVD: External ventricular drain
HCV: Hepatitis C virus
HIV: Human immunodeficiency virus
IDU: Injection drug use
LP: Lumbar puncture
MCA: Middle cerebral artery
MRI: Magnetic resonance imaging
PIIRS: Post-infectious inflammatory response syndrome
RBC: Red blood cell
TEE: Transesophageal echocardiography
WBC: White blood cell
VP: Ventriculoperitoneal
